# Two New Potential Barcodes to Discriminate *Dalbergia* Species

**DOI:** 10.1371/journal.pone.0142965

**Published:** 2015-11-16

**Authors:** Rasika M. Bhagwat, Bhushan B. Dholakia, Narendra Y. Kadoo, M. Balasundaran, Vidya S. Gupta

**Affiliations:** 1 Plant Molecular Biology Group, Biochemical Sciences Division, CSIR-National Chemical Laboratory, Pune, Maharashtra, India; 2 Forest Genetics and Biotechnology Division, Kerala Forest Research Institute, Peechi, Thrissur, Kerala, India; Biodiversity Insitute of Ontario - University of Guelph, CANADA

## Abstract

DNA barcoding enables precise identification of species from analysis of unique DNA sequence of a target gene. The present study was undertaken to develop barcodes for different species of the genus *Dalbergia*, an economically important timber plant and is widely distributed in the tropics. Ten *Dalbergia* species selected from the Western Ghats of India were evaluated using three regions in the plastid genome (*matK*, *rbcL*, *trnH-psbA*), a nuclear transcribed spacer (*nrITS*) and their combinations, in order to discriminate them at species level. Five criteria: (i) inter and intraspecific distances, (ii) Neighbor Joining (NJ) trees, (iii) Best Match (BM) and Best Close Match (BCM), (iv) character based rank test and (v) Wilcoxon signed rank test were used for species discrimination. Among the evaluated loci, *rbcL* had the highest success rate for amplification and sequencing (97.6%), followed by *matK* (97.0%), *trnH-psbA* (94.7%) and *nrITS* (80.5%). The inter and intraspecific distances, along with Wilcoxon signed rank test, indicated a higher divergence for *nrITS*. The BM and BCM approaches revealed the highest rate of correct species identification (100%) with *matK*, *matK+rbcL* and *matK*+*trnH-psb* loci. These three loci, along with *nrITS*, were further supported by character based identification method. Considering the overall performance of these loci and their ranking with different approaches, we suggest *matK* and *matK+rbcL* as the most suitable barcodes to unambiguously differentiate *Dalbergia* species. These findings will potentially be helpful in delineating the various species of *Dalbergia* genus, as well as other related genera.

## Introduction

In DNA barcoding, the sequence of a short stretch of DNA is used for accurate species identification [1], supplementing the classical taxonomic methods [2]. Although DNA barcoding has been successfully used for discriminating animal species, applying this approach for discriminating plant species is more difficult due to many challenges [3]. Plant mitochondrial genomes exhibit low rates of nucleotide substitution and high rates of chromosomal rearrangements [4], while extensive gene duplication occurs in the nuclear genome [5]. Initial DNA barcoding studies in plants have proposed a few plastid coding as well as non-coding regions, such as *rbcL* and *trnH-psbA* [6], *matK*, *rpoB*, *rpoC1* and *trnH-psbA* [7] and *atpF/H*, *matK*, *psbK/I* and *trnH-psbA* [8] as promising candidates. However, the slow evolving coding regions of plastid genomes might not possess enough variation to discriminate closely related plant species and this could lower their potential as effective barcodes [9]. This can be overcome by analyzing the selected loci either individually or in combination [10, 11]. Recently evolved nuclear region, i.e. nuclear internal transcribed spacer from ribosomal gene (*nrITS*) has also been proposed as potential barcodes [12].

*Dalbergia* Linn. F. (Family: Fabaceae) is a genus of shrubs, lianas and trees. It is confined to the tropical regions of the world with Amazonia, Madagascar, Africa and Indonesia as the centers of diversity [13, 14]. About 200 species comprise the genus, of which nearly 35 are found in India with 10–15 species in the Western Ghats (WG) alone [14, 15]. The overall species diversity is high in WG Seven species are endemic to this region (http://wgbis.ces.iisc.ernet.in/biodiversity/sahyadri_enews/newsletter/issue38/article/index.htm); hence, we choose to select WG as our study area. The *Dalbergia* genus is economically important for its quality timber. The wood of different *Dalbergia* species is used for specific purposes such as making furniture (*D*. *latifolia*, *D*. *sissoo*), boat building (*D*. *sissoo*) and manufacturing musical instruments (*D*. *melanoxylon*) [15]. Studies on tropical dry evergreen forests (TDEF) of India have indicated indiscriminate logging as one of the major factors responsible for the loss of commercial tree species, biodiversity. This is particularly the case for the species listed in Appendix II of the CITES (Convention on International Trade in Endangered Species of Wild Fauna and Flora) document [16]. The Red list of IUCN (International Union for Conservation of Nature) has more than 30 *Dalbergia* species under endangered category (http://www.iucnredlist.org) including *D*. *cochinchinensis* and *D*. *latifolia* as vulnerable species. Similarly, APFORGEN (Asia Pacific Forest Genetic Resource Programme) has identified *D*. *latifolia* as a prime concern from a conservation point of view. Moreover, as the wood of *Dalbergia* species is illegally traded in some countries, it is difficult to prove their identity and take legal action in the absence of accurate tools and methods for species identification [16]. This has facilitated fraudulent marketing and sale of poor quality wood of other tree species in place of *Dalbergia*. In this context, DNA barcoding can help as a quick way of authenticating the wood of *Dalbergia* even for legal purpose if needed.

*Dalbergia* species are morphologically variable and possess a wide range of habitat preference. This makes it difficult to classify the New World and the Old World species into natural groups [17, 18]. Over the past several decades, many revisions based on morphological characters have made the taxonomic speciation in *Dalbergia* quite challenging [12, 17, 19–23]. Moreover, very limited information is available on the molecular taxonomy of *Dalbergia* genus. There is only one report [14] describing the phylogeny of *Dalbergia* species indicating its monophyletic nature of origin. The genus was included in the evolutionary study of Leguminosae [24] to analyze the relationship of *Machaerium* and *Aeschynomene* using *trnL* and nuclear ribosomal DNA sequences [25]. Very few studies have reported on the molecular analysis of Indian *Dalbergia* species [15, 26–29], making it imperative to conduct studies on the genus on various aspects including phylogeny, diversity and end-use quality using DNA markers and sequence based polymorphism in suitable genomic regions.

In the present study, the primary focus was to develop an accurate species identification method for *Dalbergia* genus and this was addressed by developing potential DNA barcodes for the genus. We have evaluated 37 primer pairs from plastid and nuclear genomes of which four loci (*rbcL*, *matK*, *trnH-psbA* and *nrITS*) were shortlisted and various statistical parameters were employed to demonstrate their potential as barcodes to unambiguously discriminate *Dalbergia* species.

## Materials and Methods

### Ethics statement

The locations involved in the study were not part of any protected area, reserve forests or national parks except for Chinar wildlife sanctuary and Parambikulam wildlife sanctuary. The samples from these areas were collected by Kerala Forest Research Institute (KFRI), Peechi, Kerala, which is a government organization having the requisite permissions. The exact GPS coordinates for the collection sites are not available. Further, none of these species are endangered or protected species.

### Sample collection

**Fig 1:**
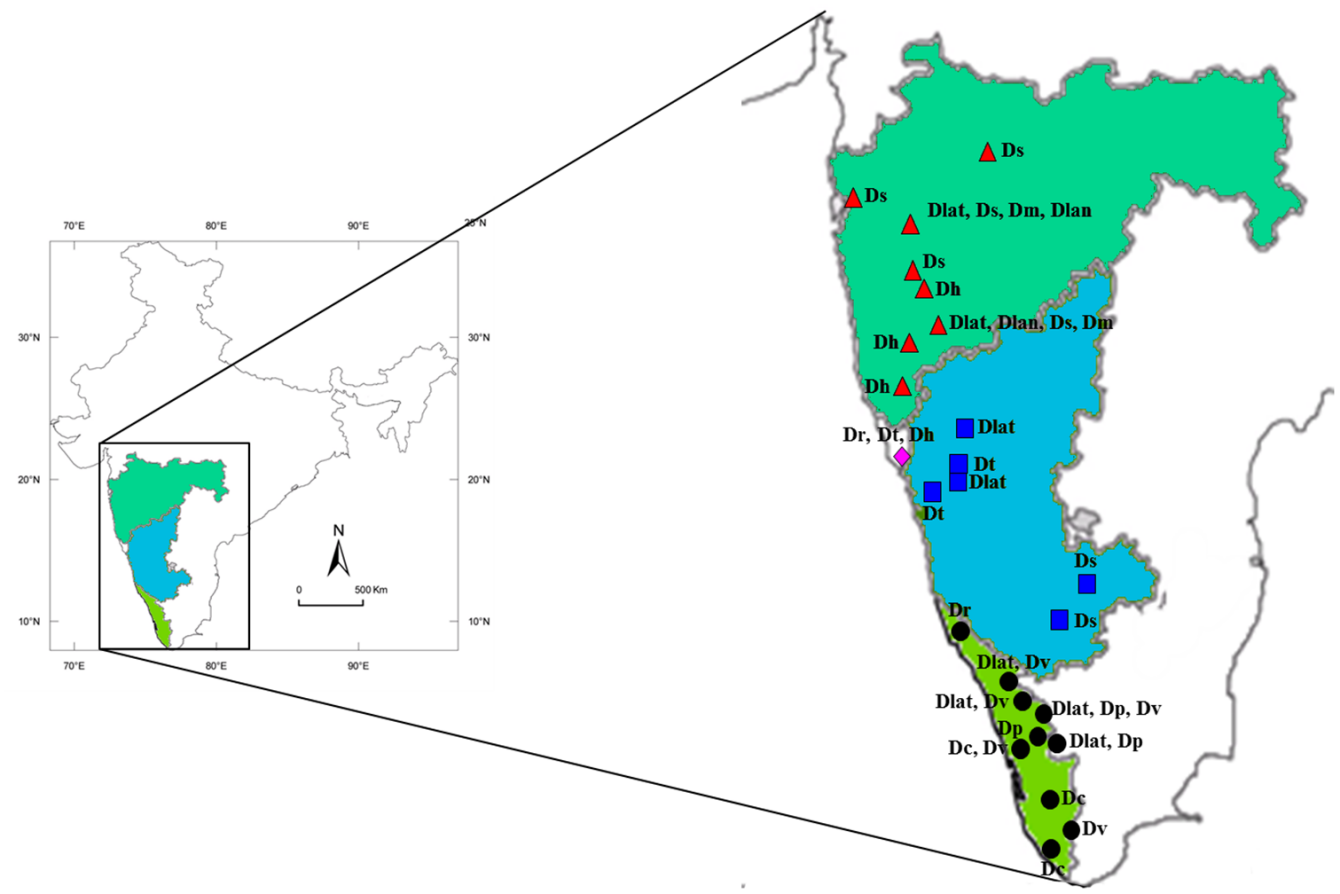
Map of India showing the locations of collection sites. The map highlights three states of India across which the Western Ghats are spread. The expended view of the inset shows the location and geographical distribution of the actual sites.

The study included 166 accessions from ten *Dalbergia* species representing three sections, section Sissoa (*Dalbergia latifolia*, *D*. *melanoxylon*, *D*. *sissoo*, *D*. *rubiginosa*, *D*. *horrida* and *D*. *tamarindifolia*), section Dalbergia (*D*. *volubilis*, *D*. *paniculata* and *D*. *lanceolaria*) [15] and section Selenolobia (*D*. *candenatensis*) [20]. We focused on the locations in WG, which is one of the most important biodiversity hotspots in India (Fig 1 and S1 Dataset). Between 5 and 25 accessions of each species were collected from different locations to understand the effect of geographical isolation on intraspecific variation in barcoding. The samples were authenticated by KFRI and the Botanical Survey of India (BSI, Western Circle, Pune, India) and the voucher specimens from each species were deposited in their respective herbaria. *Pterocarpus marsupium*, which falls outside the *Dalbergia* clade and is native to WG, was used as an out-group in the present study [14].

### DNA extraction, PCR amplification and sequencing

Total genomic DNA was extracted from fresh or dried leaf samples using the modified cetyltrimethylammonium bromide (CTAB) method [30]. At the time of initiating this study, since no specific region was recommended as universal plant barcode, based on available literature we selected the genomic loci corresponding to *matK* (7 primer pairs), *rpoC* (4 primer pairs), *rpoB* (5 primer pairs), *accD* (6 primer pairs), *ndhJ* (3 primer pairs), *ycf5* (4 primer pairs), *trnH-psbA* (5 primer pairs), *nrITS* (2 primer pairs) and *rbcL* (single primer pair) for developing the barcodes. As sequence information for most of these loci was not available for *Dalbergia* species, we attempted multiple sets of primers to amplify the respective loci from all the ten species. Thirty seven primer pairs were tested to identify the loci satisfying the set criteria for DNA barcoding. Four primer pairs (S2 Dataset) corresponding to *matK*, *rbcL*, *trnH-psbA* and *nrITS* produced highly specific amplifications (sharp bands on agarose gel) and gave good quality DNA sequences. Therefore, these were selected for further study. PCR amplifications were performed in a final volume of 20 or 25μL (S3 Dataset) and the amplicons were resolved on 1% agarose gel. Most of the PCR reactions yielded specific amplifications (i.e. sharp single bands on agarose gel) and these were directly used as templates for sequencing reactions. In the samples that generated multiple PCR products, bands corresponding to the expected size were eluted from the gel using PureLink^®^ Quick Gel Extraction Kit (Invitrogen, USA) and used as templates in sequencing reactions. Sequencing was performed using Sanger chemistry in both ends of the DNA fragment using MegaBACE DYEnamic ET dye terminator kit with MegaBACE1000 DNA Analysis System (GE Healthcare, USA).

### Sequence analysis

For each sequence, the chromatograms were inspected and poor quality 5′ and 3′ DNA sequence ends were trimmed. Post trimming lengths were maintained at least 60% of the original read length, subject to the minimum average quality score of Q20. The sequences failing this criterion were rejected and re-sequenced. All the nucleotide variations were evaluated and confirmed by aligning the chromatograms from forward and reverse sequencing results. Sequences with 70% or more overlap were considered for creating consensus sequence for each amplicon [31]. Good quality sequences from all individuals were assembled and aligned using CLUSTALW 1.83 [32]. Conserved, variable and parsimony informative sites were determined using MEGA 5.0 [33]. Distance matrices and Neighbor-Joining (NJ) trees were established in MEGA using the best fit nucleotide substitution model (chosen with AICc) [34].

### Data analysis

Genetic distance was calculated using Kimura-2-Parameter (K2P) model [35]. The interspecific divergence between the species was studied using the following three parameters: (i) average inter specific distance; (ii) average theta prime (θ'), where θ' is the mean pairwise distance within species, thus eliminating the biases associated with different individual count among species; and (iii) minimum inter specific distance. Three additional parameters were studied for the intraspecific divergence: (i) average intraspecific divergence, (ii) theta (θ) and (iii) average coalescent depth [36].

Wilcoxon signed rank tests were performed to check existence of significant divergence between the inter and intraspecific variability between the pairs of barcoding loci [11]. Consensus sequences were generated for all the ten *Dalbergia* species using TaxonDNA [37] with 1000 bootstraps. To analyze inter and intraspecific variation, sequence variants were generated with DnaSP 5.0 [38] using consensus sequences. Further, NJ trees were constructed in MEGA 5.0 with 1000 bootstraps. Based on the distance method using K2P parameter and a minimum sequence overlap of 300 bp, accurate species identification was performed by TaxonDNA or SpeciesIdentifier 1.7.7 [37] using two approaches: (i) Best match (BM) and (ii) Best close match (BCM). In these approaches, each sequence from the dataset was used as a query against the remaining sequences from the same dataset. With BM, a query sequence was identified by searching the reference sequence for the best match with the smallest genetic distance to the query. The BCM approach required a threshold value, which was calculated for each locus from pairwise summary. The threshold was a value below which 95% of all intraspecific distances were observed, leading to an upper bound value on the similarity of a barcode match [37]. If both, the query and the subject sequences were from the same species, the identification was considered as successful. Whereas, if more than one query sequence from different species exhibited equally good match, then the samples were considered as ambiguous. Another character based analysis method, Barcoding with LOGic Formulae (BLOG), was also employed [39]. This method selected the unique nucleotide position of the sequence and derived a formula to differentiate among species. It also provided concise and meaningful classification rules [40].

## Results

### Amplification success

The success rate for PCR amplification and sequencing of bidirectional reads was the highest for *rbcL* (97.6%), followed by *matK* (97.0%) and *trnH-psbA* (94.7%), while *nrITS* exhibited the lowest rate (80.5%). Nucleotide sequences of analyzed loci from all individuals were deposited in NCBI database (S1 Dataset; accession numbers—*matK*: KM276475-KM276412; *rbcL*: KM100059-KM099987; *trnH-psbA*: KM276322-KM276250 and *nrITS*: KM276165-KM276104). Using BLAST analysis, all the loci correctly identified 100% of the samples at genus level; while at species level, *nrITS* had the highest identification rate i.e. 60% followed by *rbcL* (50%), *matK* (20%) and *trnH-psbA* (10%). The low rate of species level identification might be due to the absence of species records in NCBI database and high percentage of in-dels especially in the case of *trnH-psbA* sequences.

### Nucleotide variation

The percentages of polymorphic informative (Pi) sites and variable sites were comparable for the respective loci. For *nrITS*, aligned length was 637 bp, with 29.83% sites variable and 28.89% polymorphic informative, which was the highest among all the loci (single locus as well as combination of loci). Based on the percentage of conserved sites, the most conserved loci were *rbcL* followed by *matK* and *matK+rbcL* (Table 1).

**Table 1:** Summary statistics for potential barcode loci from ten *Dalbergia* species.

Locus	*matK*	*rbcL*	*trnH*-*psbA*	*nrITS*	*matK*+ *trnH*-*psbA*	*matK*+ *rbcL*	*rbcL+ trnH*-*psbA*
No. of sequences analyzed	165	166	161	137	157	163	157
Total no. of sites	677	491	273	637	950	1168	764
Conserved sites	636 (93.94)	477 (97.15)	250 (91.58)	447 (70.17)	863 (90.84)	1113 (95.29)	724 (94.76)
Variable sites	41 (6.06)	14 (2.85)	23 (8.42)	190 (29.83)	87 (9.16)	55 (4.71)	40 (5.24)
Parsimony informative sites	40 (5.91)	14 (2.85)	15 (5.49)	184 (28.89)	55 (5.79)	54 (4.62)	29 (3.80)

**Note**: Values in parentheses are expressed in percentage.

### Inter and intraspecific divergence

#### Distance analysis and Wilcoxon signed rank test

**Fig 2:**
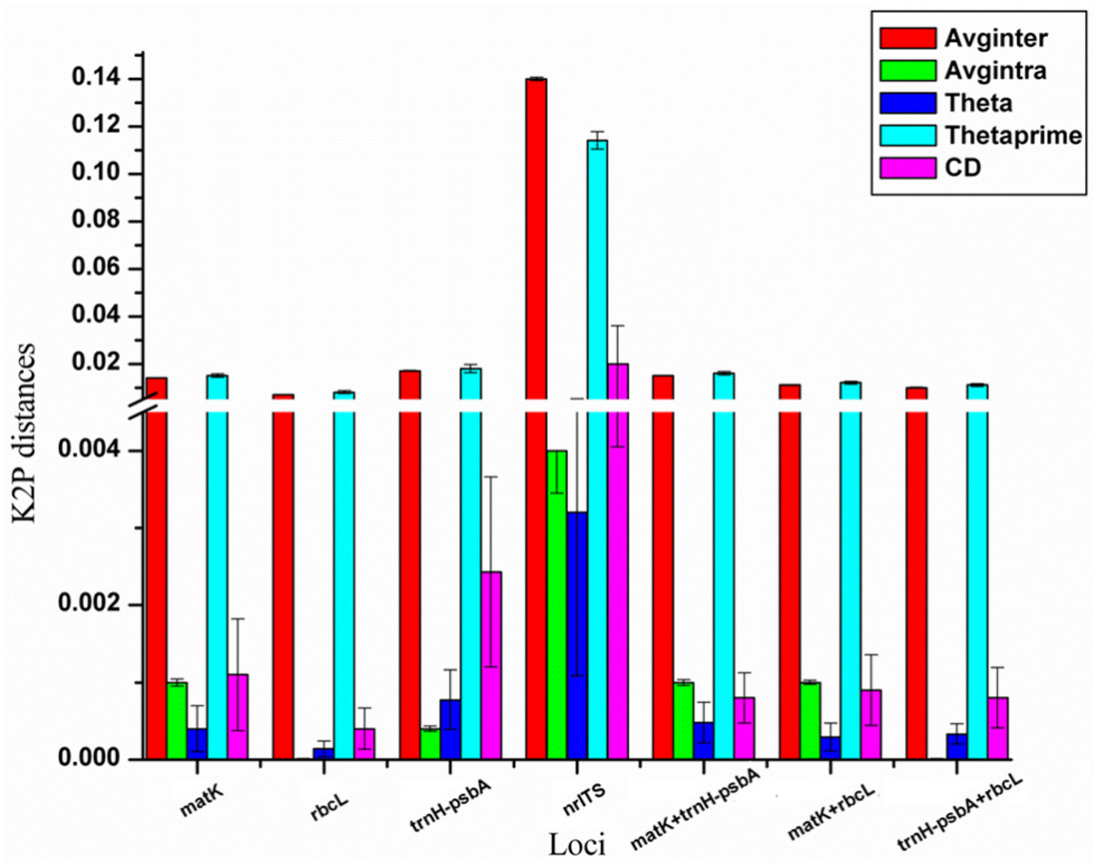
Distribution of inter and intraspecific divergence. The plot depicts inter and intraspecific divergence parameters for various loci. Avginter: Average inter specific distance, Avgintra: Average intraspecific distance, Theta, Theta prime, CD: coalescence depth.

The *nrITS* locus showed greater interspecific divergence than the plastid loci (*matK*, *rbcL* and *trnH-psbA* and their combinations) using both average inter specific distance and θ' parameters. However, in case of intraspecific divergence, *nrITS* and *rbcL* showed the highest and the lowest value, respectively. Thus, no single locus revealed the highest interspecific but the lowest intraspecific divergence (Table 2 and Fig 2). When the Wilcoxon signed rank test was used to compare the loci, *nrITS* exhibited the highest interspecific divergence followed by *trnH-psbA*, whereas *rbcL* displayed the lowest intraspecific divergence (Tables 3 and 4).

**Table 2:** Inter and intraspecific divergence values for potential barcode loci.

Distance parameters	*matK*	*rbcL*	*trnH*-*psbA*	*nrITS*	*matK*+*trnH*-*psbA*	*matK*+*rbcL*	*trnH*-*psbA*+*rbcL*
Average interspecific distance	0.014±5.74E-05	0.007±3.61E-05	0.017±1.11E-4	0.140±6.45E-4	0.015±6.45E-05	0.011±4.55E-05	0.010±5.03E-05
Theta prime (θ')	0.015±7.43E-4	0.008±5.6E-4	0.018±1.677E-3	0.114±3.656E-3	0.016±8.2E-4	0.012±5.98E-4	0.011±7.07E-4
Smallest interspecific distance	0.014±7.12E-4	0.008±5.43E-3	0.017±1.627E-3	0.156±7.922E-3	0.015±8.17E-4	0.011±5.66E-4	0.011±6.83E-4
Average intraspecific distance	0.001±4.50E-05	0.000±1.52E-05	0.000±3.52E-05	0.004±5.48E-4	0.001±3.77E-05	0.001±2.78E-05	0.000±1.50E-05
Theta (θ)	0.000±2.96E-4	0.000±1.03E-4	0.001±3.85E-4	0.003±2.112E-3	0.000±2.61E-4	0.000±1.77E-4	0.000±1.29E-4

**Table 3:** Wilcoxon signed-rank tests results for interspecific divergence of the indicated loci.

W+	W-	Inter relative ranks	Results
*matK*	*rbcL*	W+ = 85063, W- = 15, n = 412, p = 0	*matK>rbcL*
*matK*	*matK + rbcL*	W+ = 73114, W- = 39, n = 412, p = 0	*matK>matK+rbcL*
*matK*	*rbcL+trnH-psbA*	W+ = 68612, W- = 6078, n = 412, p = 0	*matK>rbcL+trnH-psbA*
*matK*	*trnH-psbA*	W+ = 29484, W- = 46370, n = 412, p = 0	*trnH-psbA>matK*
*matK*	*nrITS*	W+ = 1, W- = 85077, n = 412, p = 0	*nrITS>matK*
*matK*	*matK+trnH-psbA*	W+ = 27245, W- = 41019, n = 412, p = 0.001	*matK+trnH-psbA>matK*
*rbcL*	*nrITS*	W+ = 0, W- = 85078, n = 412, p = 0	*nrITS>rbcL*
*rbcL*	*trnH-psbA*	W+ = 5996, W- = 76625, n = 412, p = 0	*trnH-psbA>rbcL*
*rbcL*	*matK + rbcL*	W+ = 0, W- = 84255, n = 412, p = 0	*matK+rbcL>rbcL*
*rbcL*	*matK+trnH-psbA*	W+ = 0, W- = 84666, n = 412, p = 0	*matK+trnH-psbA>rbcL*
*rbcL*	*rbcL+trnH-psbA*	W+ = 1924, W- = 60204, n = 412, p = 0	*rbcL+trnH-psbA>rbcL*
*trnH-psbA*	*matK+rbcL*	W+ = 63125, W- = 17476.5, n = 412, p = 0	*trnH-psbA>matK+rbcL*
*trnH-psbA*	*matK+trnH-psbA*	W+ = 47083.5, W- = 28771.50, n = 412, p = 0	*trnH-psbA>matK+trnH-psbA*
*trnH-psbA*	*rbcL+trnH-psbA*	W+ = 73380.5, W- = 6020.5, n = 412, p = 0	*trnH-psbA>rbcL+trnH-psbA*
*trnH-psbA*	*nrITS*	W+ = 0, W- = 85078, n = 412, p = 0	*nrITS>trnH-psbA*
*nrITS*	*matK+rbcL*	W+ = 85077, W- = 1, n = 412, p = 0	*nrITS>matK+rbcL*
*nrITS*	*matK+trnH-psbA*	W+ = 85077, W- = 1, n = 412, p = 0	*nrITS>matK+trnH-psbA*
*nrITS*	*rbcL+trnH-psbA*	W+ = 85078, W- = 0, n = 412, p = 0	*nrITS>rbcL+trnH-psbA*
*matK+ rbcL*	*matK+trnH-psbA*	W+ = 37, W- = 73116, n = 412, p = 0	*matK+trnH-psbA>matK+rbcL*
*matK+ rbcL*	*rbcL+trnH-psbA*	W+ = 49627, W- = 22004, n = 412, p = 0	*matK+rbcL>rbcL+trnH-psbA*
*matK+trnH-psbA*	*rbcL+trnH-psbA*	W+ = 79759, W- = 41, n = 412, p = 0	*matK+trnH-psbA>rbcL+trnH-psbA*

**Table 4:** Wilcoxon signed-rank test results for intraspecific divergence of the indicated loci.

W+	W-	Inter relative ranks	Results
*matK*	*rbcL*	W+ = 141, W- = 12, n = 53, p = 0.002	*matK>rbcL*
*matK*	*rbcL+trnH-psbA*	W+ = 181.50, W- = 49.50, n = 53, p = 0.020	*matK>rbcL+trnH-psbA*
*matK*	*matK + rbcL*	W+ = 18.50, W- = 2.50, n = 53, p = 0.084	*matK = matK+rbcL*
*matK*	*matK+trnH-psbA*	W+ = 66, W- = 39, n = 53, p = 0.369	*matK = matK+trnH-psbA*
*matK*	*trnH-psbA*	W+ = 135.5, W- = 74.5, n = 53, p = 0.250	*matK = trnH-psbA*
*matK*	*nrITS*	W+ = 102.50, W- = 932.50, n = 53, p = 0	*nrITS>matK*
*rbcL*	*nrITS*	W+ = 10, W- = 1071, n = 53, p = 0	*nrITS>rbcL*
*rbcL*	*trnH-psbA*	W+ = 28, W- = 63, n = 53, p = 0.212	*rbcL = trnH-psbA*
*rbcL*	*rbcL+trnH-psbA*	W+ = 42, W- = 49, n = 53, p = 0.793	*rbcL+trnH-psbA = rbcL*
*rbcL*	*matK + rbcL*	W+ = 6.50, W- = 146.50, n = 53, p = 0.001	*matK+rbcL>rbcL*
*rbcL*	*matK+trnH-psbA*	W+ = 8.5, W- = 111.5, n = 53, p = 0.003	*matK+trnH-psbA>rbcL*
*trnH-psbA*	*nrITS*	W+ = 151.5, W- = 1024.5, n = 53, p = 0	*nrITS>trnH-psbA*
*trnH-psbA*	*matK+rbcL*	W+ = 80.5, W- = 129.5, n = 18, p = 0.356	*trnH-psbA = matK+rbcL*
*trnH-psbA*	*matK+trnH-psbA*	W+ = 74.5, W- = 135.5, n = 53, p = 0.250	*trnH-psbA = matK+trnH-psbA*
*trnH-psbA*	*rbcL+trnH-psbA*	W+ = 63, W- = 28, n = 53, p = 0.212	*trnH-psbA = rbcL+trnH-psbA*
*nrITS*	*matK+rbcL*	W+ = 1034, W- = 47, n = 53, p = 0	*nrITS>matK+rbcL*
*nrITS*	*matK+trnH-psbA*	W+ = 1122.5, W- = 102.5, n = 53, p = 0	*nrITS>matK+trnH-psbA*
*nrITS*	*rbcL+trnH-psbA*	W+ = 1209, W- = 16, n = 53, p = 0	*nrITS>rbcL+trnH-psbA*
*matK+rbcL*	*matK+trnH-psbA*	W+ = 42, W- = 63, n = 53, p = 0.485	*matK+trnH-psbA = matK+rbcL*
*matK+rbcL*	*rbcL+trnH-psbA*	W+ = 163, W- = 47, n = 53, p = 0.028	*matK+rbcL>rbcL+trnH-psbA*
*matK+trnH-psbA*	*rbcL+trnH-psbA*	W+ = 167.50, W- = 22.50, n = 53, p = 0.002	*matK+trnH-psbA>rbcL+trnH-psbA*

#### Barcode gap

**Fig 3:**
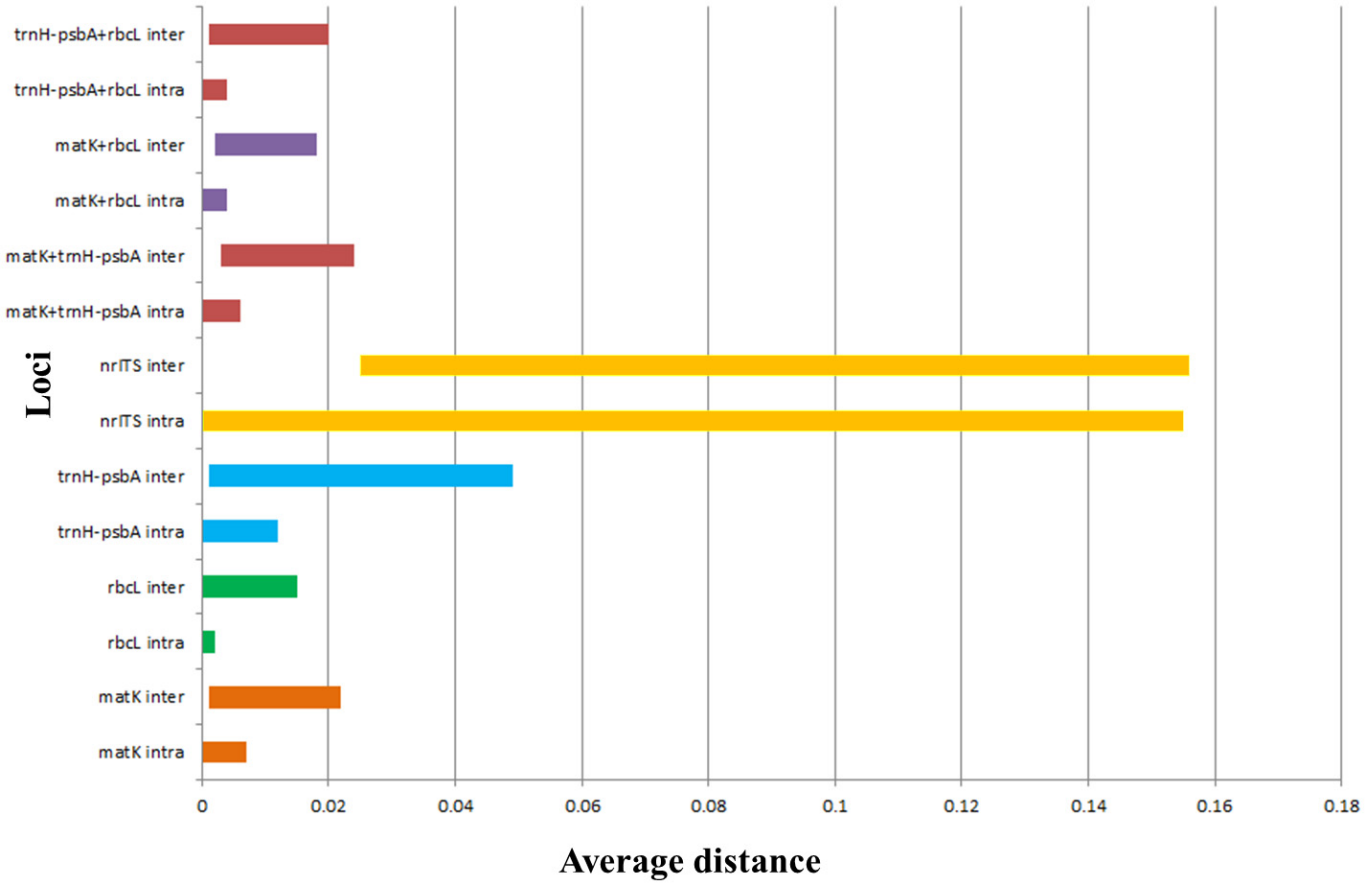
The barcoding gap. Graph of smallest interspecific and largest intraspecific distances highlighting the overlapping divergence.

Barcode gap represents the absence of overlapping regions between inter and intraspecific distances. The barcode gap was absent for all the marker loci used in the present study, indicating overlaps between inter and intraspecific distances (Fig 3). However, the mean interspecific divergence was significantly higher than that of the corresponding intraspecific divergence for each of the loci. This was further confirmed by analysis carried out using TaxonDNA.

#### Tree based analyses

**Fig 4:**
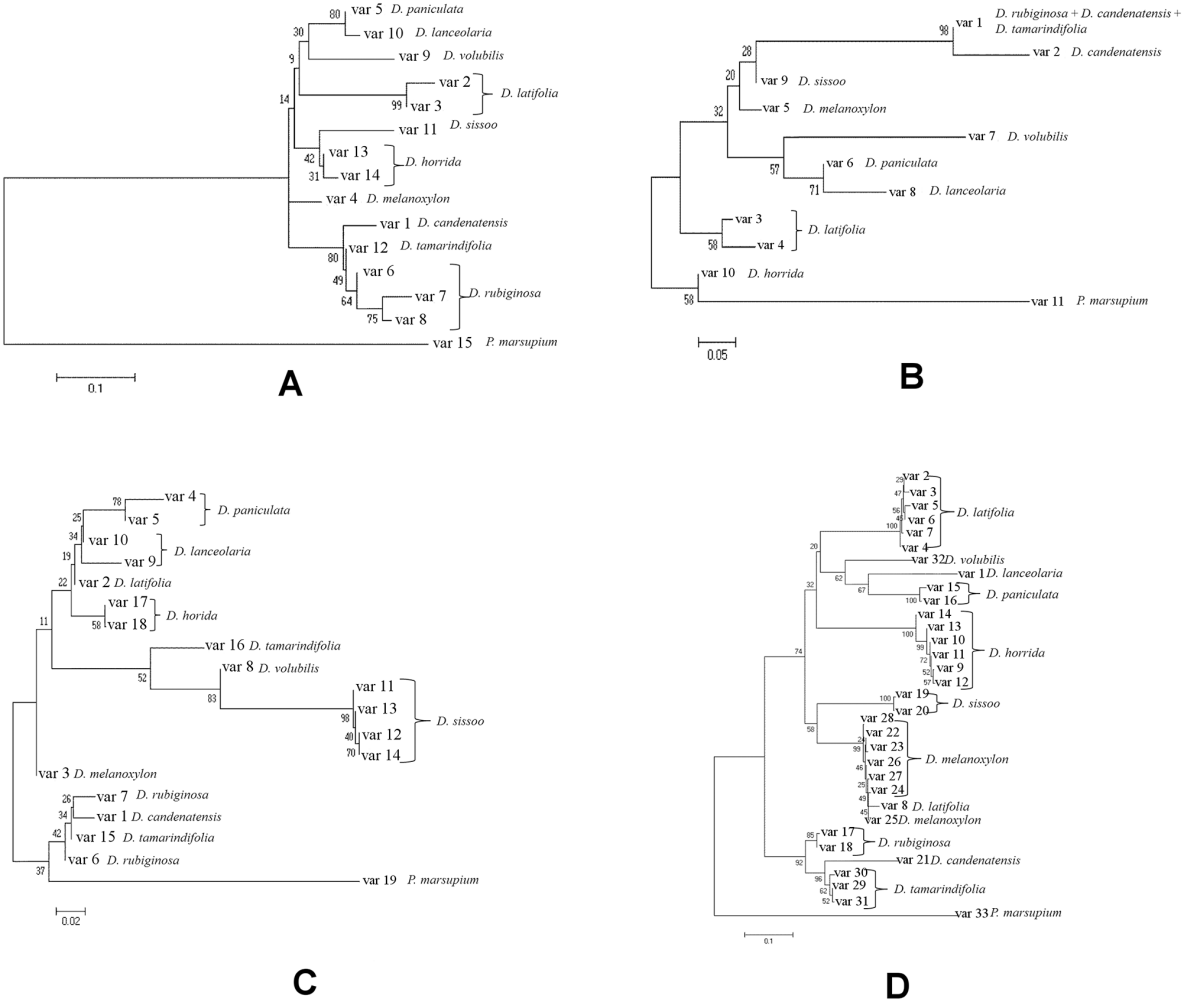
Single locus NJ trees. NJ trees were constructed using MEGA 5.0 based on K2P distance model–**A,** *matK*; **B,** *rbcL*; **C:** *trnH-psbA*, **D,** *nrITS*.

**Fig 5:**
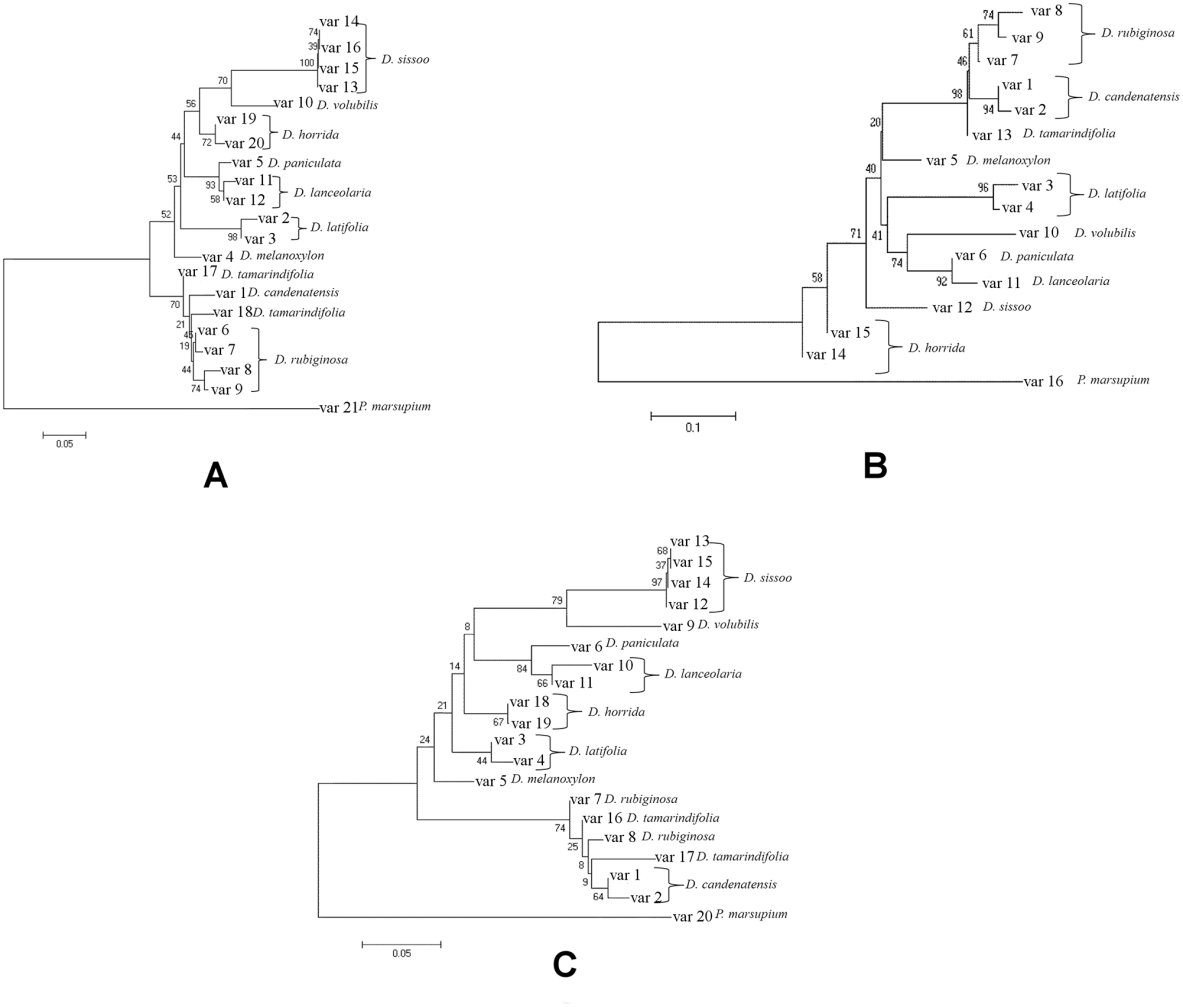
NJ trees with combined loci. NJ trees constructed using MEGA 5.0 based on K2P distance model–**A,** *matK+rbcL*; **B,** *matK+trnH-psbA*; **C,** *rbcL+ trnH-psbA*.

The sequence variants of each marker locus were determined using DnaSP 5.0 and MEGA 5.0 as mentioned previously. Among all loci, *nrITS* exhibited the maximum number of sequence variants (Table 5). By including all the sequence variants, seven NJ trees were constructed with *matK*, *rbcL*, *trnH-psbA* and *nrITS* either alone (Fig 4) or in combinations (Fig 5). All of them except *rbcL* revealed a separate cluster for each species and *rbcL* could not differentiate between *D*. *rubiginosa*, *D*. *candenatensis* and *D*. *tamarindifolia*. Interestingly, except *trnH-psbA* all other loci (*matK*, *rbcL*, *nrITS* and *matK+rbcL*) either alone or in combination were capable of grouping together all three species-clusters from the section Dalbergia (*D*. *volubilis*, *D*. *lanceolaria* and *D*. *paniculata*). This agrees with a previous report on genome size variation and evolution of *Dalbergia* species which found that *D*. *lanceolaria* and *D*. *paniculata* were closely related [15]. These observations indicated that *matK*, *nrITS*, *rbcL* and *matK+rbcL* could correctly identify the reported relationships among the *Dalbergia* species and hence, they could most likely be successful as barcodes for this genus.

**Table 5:** Distribution of sequence variants among the ten *Dalbergia* species across all loci.

Species	Number of sequence variants						
	*matK*	*rbcL*	*trnH-psbA*	*nrITS*	*matK*+*rbcL*	*matK*+*trnH-psbA*	*rbcL*+*trnH-psbA*
Dc	1	2	1	1	2	1	2
Dlat	2	2	1	7	2	2	2
Dm	1	1	1	7	1	1	1
Dp	1	1	2	2	1	1	1
Dr	3	1	2	2	3	4	2
Dv	1	1	1	1	1	1	1
Dlan	1	1	2	1	1	2	2
Ds	1	1	4	2	1	4	4
Dt	1	1	2	3	1	2	2
Dh	2	1	2	6	2	2	2

**Species codes**:—Dc: *D*. *candenatensis*, Dlat: *D*. *latifolia*, Dm: *D*. *melanoxylon*, Dp: *D*. *paniculata*, Dr: *D*. *rubiginosa*, Dv: *D*. *volubilis*, Dlan, *D*. *lanceolaria*, Ds: *D*. *sissoo*, Dt: *D*. *tamarindifolia*, Dh: *D*. *horrida*.

#### Similarity based approach

To evaluate the accuracy of these potential barcodes in species assignments, the BM and BCM parameters from TaxonDNA analysis were used (Table 6). Finding a standard threshold for BCM approach is difficult as there is a large variation in inter and intraspecific divergence across all loci in different plant systems [9]. Moreover, our approach to use multiple accessions of each species, as suggested by Pettengill and Neel [9] has ensured that the basic requirement was fulfilled and therefore, we chose to use calculated thresholds. The calculated threshold value per locus varied from 0.12% in *rbcL*+*trnH*-*psbA* to 1.2% in *nrITS*. With the BM and BCM approaches, the success rate of correct identification was unambiguously 100% for *matK*, *matK+trnH-psbA* and *matK+rbcL* and 0% incorrect identification (Table 6).

**Table 6:** Results from similarity based analysis using TaxonDNA.

Regions	Best Match			Best Close Match				
	Correct	Ambiguous	Incorrect	Correct	Ambiguous	Incorrect	Sequence without any match closer than threshold	Threshold (%)
*matK*	165 (100.00)	0 (0.00)	0 (0.00)	165 (100.00)	0 (0.00)	0 (0.00)	0 (0.00)	0.74
*rbcL*	126 (75.90)	40 (24.09)	0 (0.00)	126 (75.90)	40 (24.09)	0 (0.00)	0 (0.00)	0.20
*trnH*-*psbA*	109 (67.70)	51 (31.67)	1 (0.62)	108 (67.08)	51 (31.67)	0 (0.00)	2 (1.24)	0.37
*nrITS*	134 (99.30)	0 (0.00)	1 (0.73)	135 (99.26)	0 (0.00)	1 (0.73)	0 (0.00)	1.20
*matK*+ *trnH*-*psbA*	157 (100.00)	0 (0.00)	0 (0.00)	157 (100.00)	0 (0.00)	0 (0.00)	0 (0.00)	0.52
*matK*+ *rbcL*	163 (100.00)	0 (0.00)	0 (0.00)	163 (100.00)	0 (0.00)	0 (0.00)	0 (0.00)	0.25
*rbcL+trnH*-*psbA*	135 (85.98)	21 (13.37)	1 (0.63)	135 (85.98)	21 (13.37)	0 (0.00)	1 (0.63)	0.12

**Note**: Values in parentheses are expressed in %.

#### Character based approach

The data analysis resulted into logic formulae as well as revealed information regarding correctly classified, wrongly classified and not classified species. Only the analysis done using *matK*, *nrITS*, *matK+rbcL* and *matK+trnH-psbA* loci could assign the characteristic nucleotide positions for all the species with 100% correct classification (Table 7).

**Table 7:** Character based approach for species identification in *Dalbergia*.

Locus	cc	wc	nc	Formula									
				Dc	Dlat	Dm	Dp	Dr	Dv	Dlan	Ds	Dt	Dh
*matK*	100	0	0	362 = A	84 = T	206 = A	28 = G, 166 = T, 368 = G	440 = G	7 = C	166 = A	51 = G	292 = T, 362 = T, 440 = T	368 = G, 422 = A
*rbcL*	76.56	0	23.44	339 = A	191 = T	19 = T	35 = T, 186 = G	-	485 = T	35 = C	19 = A, 179 = A, 86 = A, 191 = C, 458 = G, 485 = C	-	458 = T
*trnH-psbA*	69.35	0	30.65	24 = T	-	12 = C	118 = G	139 = A	52 = C114 = A, 228 = A	26 = T	114 = G, 228 = A	52 = A, 228 = A	114 = G, 228 = G
*nrITS*	100	0	0	107 = C	122 = C, 456 = T	83 = C	621 = A	132 = T,231 = G	43 = C	132 = T, 456 = C	128 = A	107 = G	539 = C, 637 = C
*matK+rbcL*	100	0	0	1052 = T	711 = T	697 = A	95 = A, 657 = T, 711 = G, 1011 = A	931 = G OR 783 = A	1011 = G	657 = A	1018 = G	783 = T, 801 = A, 931 = T, 1052 = C	922 = T
*matK+trnH-psbA*	100	0	0	362 = A	84 = T	206 = A	795 = G	440 = G OR 292 = A	422 = C,905 = A	166 = A	422 = A, 905 = A	292 = T, 362 = T, 440 = T	422 = A, 905 = G
*rbcL+trnH-psbA*	86.99	0	13.01	515 = T	191 = T	503 = C	609 = G	630 = A	495 = T, 734 = C	95 = A, 191 = C, 734 = A	495 = G	-	191 = C, 503 = A, 686 = A, 734 = A

**Note**: cc: correctly classified, wc: wrongly classified, nc: not classified

**Species codes**:- Dc: *D*. *candenatensis*, Dlat: *D*. *latifolia*, Dm: *D*. *melanoxylon*, Dp: *D*. *paniculata*, Dr: *D*. *rubiginosa*, Dv: *D*. *volubilis*, Dlan, *D*. *lanceolaria*, Ds: *D*. *sissoo*, Dt: *D*. *tamarindifolia*, Dh: *D*. *horrida*.

### Overall performance of the loci

The different parameters used for screening potential barcode loci were ranked based on their performance on a scale of 1–10. In case of NJ trees, the ranking was done based on clustering of the species. Those loci which separated all the species irrespective of intraspecific variation were given ten marks, while for the remaining loci, the scale was determined based on the number of species clubbed together. For inter- and intraspecific distances, the difference between the maximum and minimum distance was calculated to determine the scale for each locus. For BM and BCM methods, the percent values corresponding to correct, ambiguous and incorrect classification were used to rank the loci. A similar methodology was also applied for BLOG. Finally, for Wilcoxon signed rank test, the locus which performed the best in a pair in both, inter and intraspecific distance determinations, was ranked the highest (Table 8).

**Table 8:** Comparative ranking of loci used in DNA barcoding of *Dalbergia*.

Parameters	*matK*	*rbcL*	*trnH-psbA*	*nrITS*	*matK+rbcL*	*matK+ trnH-psbA*	*rbcL+ trnH-psbA*
Barcode	10	7	8	8	10	8	8
Inter and intra specific distances	10	7	8	8	10	8	8
Best match and best close match	10	7.2	4.9	6.6	10	10	3.3
BLOG	10	7	7	7.5	10	8.6	6.4
Wilcoxon Signed Rank test	4	5	9	5.5	3.5	5	6
**Total**	**44**	**33.2**	**36.9**	**35.6**	**43.5**	**39.6**	**31.7**

**Note**: Larger values indicate better performance.

## Discussion

Paul Hebert’s research in 2003 on species identification using short stretches of DNA from a well characterized region of the genome, gave birth to the concept of DNA barcoding [41]. Initial efforts proved the reliability of mitochondrial cytochrome c oxidase 1 (*cox1*) gene as an impressive barcode in animals [42]. However, initial research on plant DNA barcoding suggested that species discrimination in plants with a single universal locus is difficult. This is primarily due to various phenomena such as polyploidy, hybridization, heteroplasy etc., which result in the formation of continuous range of variable characters and making delineation a difficult task. Alternatively, sufficient time is often required to accumulate mutations in organisms which are responsible for separation of closely related species. However, the lack of such sufficient genetic variation hampers species level discrimination of plants by DNA barcoding [8]. This problem is exaggerated in woody plants because of longer generation time and lower mutation rate. It is also difficult to differentiate species in taxonomically complex groups where species are narrowly defined. Additionally, large ancestral population sizes and low levels of within species gene flow for plastid markers create difficulty in barcode based identification [3, 8]. In order to resolve these problems, several attempts have been made to establish DNA barcodes using multiple genes from different plant genomes for specific families such as Myristicaceae [43], Lemnaceae [44], Zingiberaceae [45], Podocarpaceae [46] or genera such as *Paeonia* [47], *Acacia* [48], *Paphiopedilum* [49], *Parnassia* [50] and *Gossypium* [51]. However, from different studies, it appears that finding a universal barcode or even a barcode at family level is difficult and it may be possible to establish a discriminating barcode only at genus level [52].

There are few reports on DNA barcoding of tropical tree species [16, 31, 53] which include Amazonian as well as Indian forest trees. These studies have used *nrITS*, *matK*, *rbcL* and *trnH-psbA* loci. However, there are scanty reports on DNA barcoding of trees exclusively from WG of India. A study on 143 tree species from tropical dry evergreen forests in India covering 114 genera and 42 families revealed that combination of *matK* and *rbcL* loci gave the highest success in accurate identification [16]. Similarly, DNA barcoding of medicinal plants from the family Fabaceae revealed 80% and 96% success at species and genus level, respectively using *matK* locus, while the *ITS2* locus gave more than 80% success at species level and 100% success at genus level [54]. However, none of the above mentioned studies included *Dalbergia*. A recent study on tropical tree species from India (149 species from 82 genera and 38 families) included three *Dalbergia* species and suggested that *ITS* and *trnH-psbA* might not be highly successful [31]. Efforts to resolve the sister species complex of *Acacia* from Fabaceae using *rbcL*, *trnH-psbA* (same primer sequence as we have used in our study) and *matK* recommended all the three regions for barcoding [48]. On the contrary, studies on *Aspalathus* using *ITS* (different primers than the ones used in our study), *psbA-trnH* and *trnT-trnL* concluded that all the three loci were unable to resolve the species [55]. It was observed that the output from *matK* analysis was variable based on the plant systems as well as on the combination of primers used for analysis. However, the Consortium for the Barcode of Life (CBOL) proposed 90% success with *matK* for plants. Our study also identified *matK* as one of the potential loci for DNA barcoding. Thus, *matK*, *nrITS* and *rbcL* individually or in their combinations could be explored as the potential DNA barcodes in various plant genera [53].

### Assessment of the four candidate barcodes in *Dalbergia* genus

In the present study, the amplification and sequencing success rate in *Dalbergia* ranged from 80.5% (for *nrITS*) to 97.6% (for *rbcL*). While the *rbcL* locus was reported to be easy to amplify and sequence across a broad range of plant taxa, but offers low species resolution, the rapidly evolving *matK*, locus, is known for its high discriminatory power with low universality [56]. Hence, the *matK* is popular for species discrimination in case of angiosperms [3]. However, mixed results ranging from high success rate [56, 57] to poor discrimination [3, 11] have been reported for *matK*. Even in the present study, *matK* showed good resolving power and although *trnH-psbA* showed good universality and higher discrimination, it also has variable length, presence of homopolymers, inversions and insertion of *rps19* gene [58–60]. Similarly, while the *nrITS* locus is a commonly used nuclear marker for phylogenetic studies [5], it was, however, not preferred for barcoding studies initially because of fungal contamination, paralogous gene copies and problems in recovery [8]. In our study, similarity search using BLAST did not reveal any problem of fungal contamination in *nrITS* sequences; however, the sequencing success was low (80%), which might be due to the presence of divergent gene copies as reported earlier [5]. In case of *trnH-psbA* which gave 94.7% sequencing success, our data revealed the presence of T and A repeats, without any insertion of *rps19* gene when checked by BLAST.

The overall interspecific distances were high compared to intraspecific distances and no significant barcode gap was observed in the present study. Usually in the closely related plant species, plastid regions such as *rbcL* and *matK* do not generate a barcode gap [57]. Several studies have also revealed the absence of barcode gap in different plant systems such as *Agalinis* [9], *Parnassia* [50], *Gossypium* [51] medicinal plants [12] and *Dioscorea* [61]. Furthermore in the NJ tree based analysis, *nrITS*, *matK* and *trnH-psbA* and their combinations formed separate clusters for each species. However, *rbcL* could not differentiate *D*. *rubiginosa*, *D*. *candenatensis* and *D*. *tamarindifolia*, which could be because of the conserved nature of the gene [62]. Similar behavior of *rbcL* was also reported in *Carex* [58]. Together this suggested that individually *rbcL* might not serve as a good barcode but can be utilized in combination with other loci.

A recent report on DNA barcoding of eight *Dalbergia* species from Vietnam recommended *ITS* locus as a potential barcode based on UPGMA analysis and nucleotide diversity [63]. It has been reported that being a multigene family, 18s-26s rDNA is subjected to concerted evolution. In certain cases, *ITS1* [64, 65] and *ITS2* [12, 60, 65, 66] have been used as separate loci for DNA barcoding. However, point mutations displayed by *ITS1* and *ITS2* also contribute to high intraspecific variations [67]. We used the complete ITS region (ITS1-5.8S-ITS2) as a single barcoding locus. In our study, *nrITS* showed high intraspecific variation with high species discrimination, leading to incorrect identification with BM and BCM. However, DNA barcoding of eight *Dalbergia* species from Vietnam [63], did not use the species from the current study. A reanalysis of the data from NCBI for the species used in the Vietnam study along with dataset from our study revealed a high number of sequence variants for most of the species (S1 Fig). Moreover, from the available sequence data in NCBI for the Vietnam study [63], we could find only one *nrITS* sequence each for *D*. *dialoides*, *D*. *entadoides* and *D*. *hancei* making it difficult to assay the intraspecific variation. It was therefore, not possible to comment on either the intraspecific diversity of these species, which is an important factor for DNA barcoding or the suitability of *nrITS* as the potential barcode for *Dalbergia* species. It is essential to sample enough number of accessions for each of these species, ideally from different geographical locations, to sample the intraspecific variation from the entire distributional range [53].

## Conclusions

In the present study 7–26 accessions of ten *Dalbergia* species each collected from different geographic locations in WG region of India were screened using 37 primer pairs from nuclear and plastid genes. Four loci (*rbcL*, *matK*, *trnH-psbA* and *nrITS*) and their combinations were further evaluated with five different analyses and ranked based on their performance. These studies have revealed *matK* and *matK+rbcL* loci as the most suitable barcodes to discriminate *Dalbergia* species.

## Supporting Information

S1 Fig NJ tree. Combined analysis of *nrITS* sequences submitted by Phong et al. [63] with those generated in this study, revealing high intraspecific variation and several sequence variants for most species.
(TIF)

S2 Fig NJ tree. Representative tree for *matK+rbcL* using all the individuals without any division. Dc: *D*. *candenatensis*, Dlat: *D*. *latifolia*, Dm: *D*. *melanoxylon*, Dp: *D*. *paniculata*, Dr: *D*. *rubiginosa*, Dv: *D*. *volubilis*, Dlan: *D*. *lanceolaria*, Ds: *D*. *sissoo*, Dt: *D*. *tamarindifolia*, Dh: *D*. *horrida*.
(TIF)

S1 Dataset Sample details. List of all samples with collection details and GenBank accession numbers.
(DOCX)

S2 Dataset Primer details. Primers used in DNA barcoding of *Dalbergia* species.
(DOCX)

S3 Dataset PCR reaction details. PCR conditions for *matK*, *rbcL*, *trnH-psbA* and *nrITS*
(DOCX)
