# Supplementary figures and images for: Two New Potential Barcodes to Discriminate Dalbergia Species
Source: PLoS One. 2015 Nov 16;10(11):e0142965. doi: 10.1371/journal.pone.0142965 (PMC4646644; doi:10.1371/journal.pone.0142965)

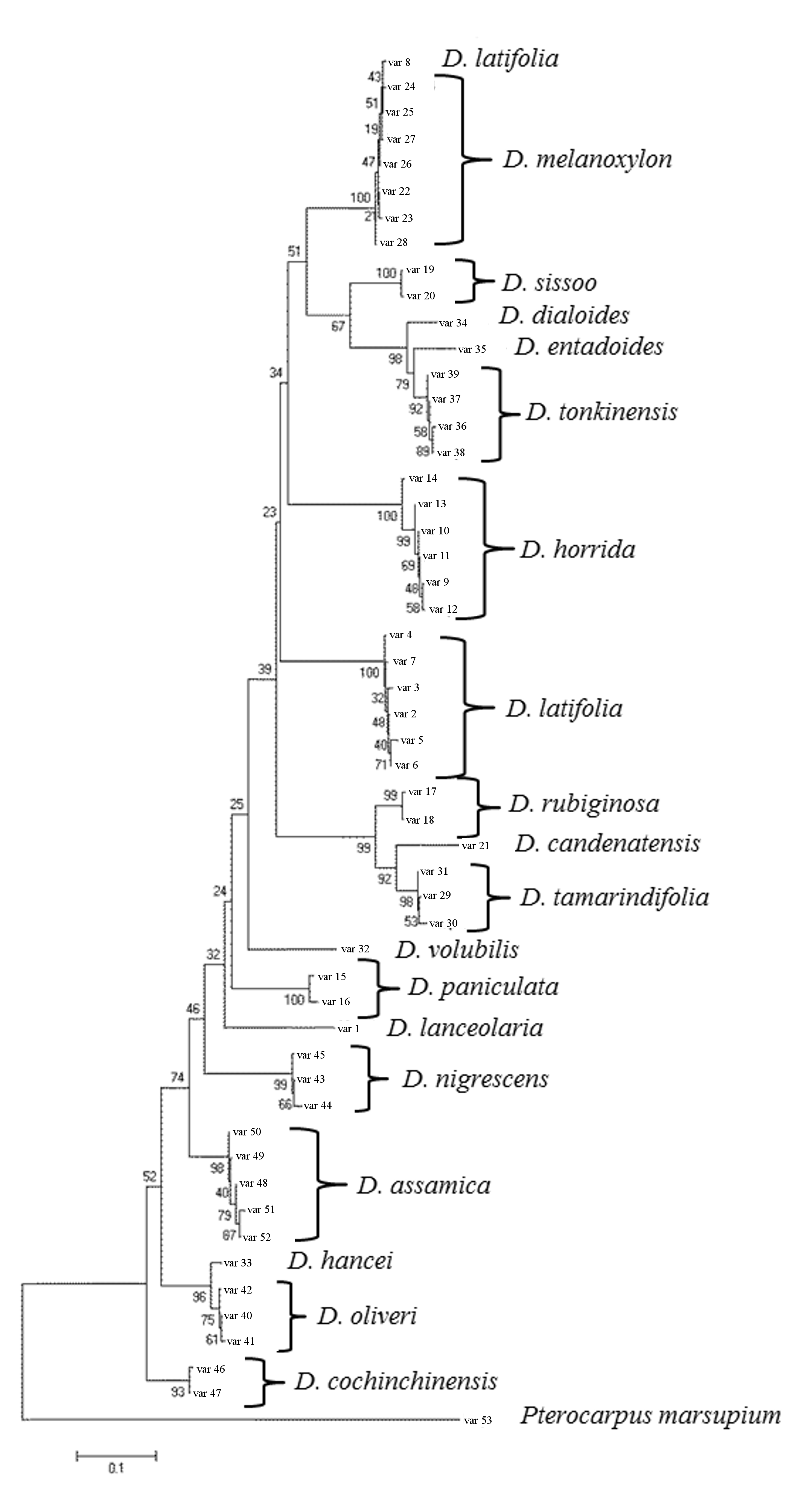

Supplement: S1 Fig — Combined analysis of nrITS sequences submitted by Phong et al. [63] with those generated in this study, revealing high intraspecific variation and several sequence variants for most species. (TIF) [file pone.0142965.s001.tif]
