# Supplementary material for: Two New Potential Barcodes to Discriminate Dalbergia Species
Source: PLoS One. 2015 Nov 16;10(11):e0142965. doi: 10.1371/journal.pone.0142965 (PMC4646644; doi:10.1371/journal.pone.0142965)
Supplement: S1 Dataset — List of all samples with collection details and GenBank accession numbers. (DOCX) [file pone.0142965.s003.docx]

**S1 Dataset**. **Sample details.** List of all samples with collection details and GenBank accession numbers

| **Sr No.** | **Accession** | **Locality** | **GenBank accession number** | | | |
| --- | --- | --- | --- | --- | --- | --- |
|  |  |  | ***matK*** | ***rbcL*** | ***trnH-psbA*** | ***nrITS*** |
|  | *D. rubiginosa*1 | Methunganam, Kasargod, Kerala | KM276475 | KM100059 | KM276322 | KM276165 |
|  | *D. rubiginosa*2 | Methunganam, Kasargod, Kerala | KM276476 | KM100060 | KM276323 | - |
|  | *D. rubiginosa*3 | Methunganam, Kasargod, Kerala | KM276477 | KM100061 | KM276324 | - |
|  | *D. rubiginosa*4 | Methunganam, Kasargod, Kerala | KM276478 | - | - | KM276166 |
|  | *D. rubiginosa*5 | Methunganam, Kasargod, Kerala | KM276479 | KM100062 | KM276325 | KM276167 |
|  | *D. rubiginosa*6 | Methunganam, Kasargod, Kerala | KM276480 | KM100063 | KM276326 | KM276168 |
|  | *D. rubiginosa*7 | Methunganam, Kasargod, Kerala | KM276481 | KM100064 | KM276327 | KM276169 |
|  | *D. rubiginosa*8 | Methunganam, Kasargod, Kerala | KM276482 | KM100065 | KM276328 | KM276170 |
|  | *D. rubiginosa*9 | Methunganam, Kasargod, Kerala | KM276483 | KM100066 | KM276329 | KM276171 |
|  | *D. rubiginosa*10 | Methunganam, Kasargod, Kerala | KM276484 | KM100067 | KM276330 | KM276172 |
|  | *D. rubiginosa*11 | Thekkal, Kasargod, Kerala | KM276485 | KM100068 | KM276331 | KM276173 |
|  | *D. rubiginosa*12 | Thekkal, Kasargod, Kerala | KM276486 | KM100069 | KM276332 | KM276174 |
|  | *D. rubiginosa*13 | Thekkal, Kasargod, Kerala | KM276487 | KM100070 | KM276333 | KM276175 |
|  | *D. rubiginosa*14 | Thekkal, Kasargod, Kerala | KM276488 | KM100071 | KM276334 | KM276176 |
|  | *D. rubiginosa*15 | Thekkal, Kasargod, Kerala | KM276489 | KM100072 | KM276335 | KM276177 |
|  | *D. rubiginosa*16 | Thekkal, Kasargod, Kerala | KM276490 | KM100073 | KM276336 | KM276178 |
|  | *D. rubiginosa*17 | Thekkal, Kasargod, Kerala | - | KM100074 | KM276337 | - |
|  | *D. rubiginosa*18 | Thekkal, Kasargod, Kerala | KM276491 | KM100075 | KM276338 | KM276179 |
|  | *D. rubiginosa*19 | Thekkal, Kasargod, Kerala | KM276492 | KM100076 | KM276339 | KM276180 |
|  | *D. rubiginosa*20 | Thekkal, Kasargod, Kerala | KM276493 | KM100077 | KM276340 | KM276181 |
|  | *D. rubiginosa* 21 | Anshighat, Karnataka-Goa border | KM276494 | KM100078 | KM276341 | KM276182 |
|  | *D. rubiginosa* 22 | Anshighat, Karnataka-Goa border | KM276495 | KM100079 | KM276342 | KM276183 |
|  | *D. candenatensis* 1 | Kollam, Kerala | KM276387 | KM099961 | KM276226 | KM276089 |
|  | *D. candenatensis* 2 | Kollam, Kerala | - | KM099962 | KM276227 | KM276090 |
|  | *D. candenatensis* 3 | Kollam, Kerala | KM276388 | KM099963 | KM276228 | - |
|  | *D. candenatensis* 5 | Kollam, Kerala | KM276389 | KM099964 | KM276229 | - |
|  | *D. candenatensis* 6 | Alleppy, Kerala | KM276390 | KM099965 | KM276230 | KM276091 |
|  | *D. candenatensis* 7 | Alleppy, Kerala | KM276391 | KM099966 | KM276231 | KM276092 |
|  | *D. candenatensis* 8 | Cherai, Paravoor, Ernakulam, Kerala | KM276392 | KM099967 | KM276232 | KM276093 |
|  | *D.* candenatensis 9 | Cherai, Paravoor, Ernakulam, Kerala | KM276393 | KM099968 | KM276233 | - |
|  | *D. candenatensis* 11 | Anachal, Paravoor, Ernakulam, Kerala | KM276394 | KM099969 | KM276234 | - |
|  | *D. candenatensis* 13 | Anachal, Paravoor, Ernakulam, Kerala | KM276395 | KM099970 | KM276235 | - |
|  | *D. candenatensis* 14 | Anachal, Paravoor, Ernakulam, Kerala | KM276396 | KM099971 | - | - |
|  | *D. candenatensis* 15 | Anachal, Paravoor, Ernakulam, Kerala | KM276397 | KM099972 | KM276236 | - |
|  | *D. candenatensis* 17 | Anachal, Paravoor, Ernakulam, Kerala | KM276398 | KM099973 | KM276237 | - |
|  | *D. candenatensis* 18 | Anachal, Paravoor, Ernakulam, Kerala | KM276399 | KM099974 | - | KM276094 |
|  | *D. candenatensis* 19 | Anachal, Paravoor, Ernakulam, Kerala | KM276400 | KM099975 | KM276238 | - |
|  | *D. candenatensis* 20 | Anachal, Paravoor, Ernakulam, Kerala | KM276401 | KM099976 | KM276239 | - |
|  | *D. candenatensis* 21 | Anachal, Paravoor, Ernakulam, Kerala | KM276402 | KM099977 | KM276240 | - |
|  | *D. candenatensis* 22 | Anachal, Paravoor, Ernakulam, Kerala | KM276403 | KM099978 | KM276241 | KM276095 |
|  | *D.* latifolia 2 | Near Malabar cement factory, Walayar, Palakkad, Kerala | KM276413 | KM099995 | - | KM276112 |
|  | *D. latifolia* 3 | Near Malabar cement factory, Walayar, Palakkad, Kerala | KM276414 | KM099996 | KM276258 | KM276113 |
|  | *D. latifolia* 4 | Near Malabar cement factory, Walayar, Palakkad, Kerala | KM276415 | KM099997 | KM276259 | KM276114 |
|  | *D. latifolia* 6 | Mangalam Dam Palakkad, Kerala | KM276416 | KM099998 | KM276260 | - |
|  | *D. latifolia* 9 | Mukkali, Attappady, Palakkad, Kerala | KM276417 | KM099999 | KM276261 | KM276115 |
|  | *D. latifolia* 11 | Chinnar Wildlife Sanctuary, Kerala | KM276418 | KM100000 | KM276265 | - |
|  | *D. latifolia* 21 | Chinnar Wildlife Sanctuary, Kerala | KM276419 | KM100001 | KM276266 | - |
|  | *D. latifolia* 31 | Chinnar Wildlife Sanctuary, Kerala | KM276420 | KM100002 | KM276267 | - |
|  | *D. latifolia* 21A | Kurichiad, Wayanad, Kerala | KM276421 | KM100003 | KM276262 | - |
|  | *D. latifolia* 22 | Kurichiad, Wayanad, Kerala | KM276422 | KM100004 | KM276263 | KM276116 |
|  | *D. latifolia* 23 | Kurichiad, Wayanad, Kerala | KM276423 | KM100005 | KM276264 | - |
|  | *D. latifolia* B | Gadhinglaj, Dist. Kolhapur, Maharashtra | KM276424 | KM100006 | KM276268 | KM276117 |
|  | *D. latifolia* D | Radhanagari, Maharashtra | KM276425 | KM100007 | KM276269 | KM276118 |
|  | *D. latifolia* E | Radhanagari, Maharashtra | KM276426 | KM100008 | KM276270 | KM276119 |
|  | *D. latifolia* F | Kerle, Kolhapur, Maharashtra | KM276427 | KM100009 | KM276271 | - |
|  | *D. latifolia* G | Panhala, Kolhapur, Maharashtra | KM276428 | KM100010 | KM276272 | KM276120 |
|  | *D. latifolia* H | TabakUdyan, Panhala, Kolhapur, Maharashtra | KM276429 | KM100011 | KM276273 | - |
|  | *D. latifolia* I | Jotiba, Kolhapur, Maharashtra | KM276430 | KM100012 | KM276274 | KM276121 |
|  | *D. latifolia* J | Jotiba, Kolhapur, Maharashtra | KM276431 | KM100013 | KM276275 | KM276122 |
|  | *D. latifolia* K | Kolhapur, Maharashtra | KM276432 | KM100014 | KM276276 | KM276123 |
|  | *D. latifolia* L | Karnataka | KM276433 | KM100015 | KM276277 | KM276124 |
|  | *D. latifolia*P | Karnataka | KM276434 | KM100016 | KM276278 | KM276125 |
|  | *D. latifolia*Q | Pirangut, Pune, Maharashtra | KM276435 | KM100017 | KM276279 | KM276126 |
|  | *D. latifolia* R | University of Agricultural Sciences, Dharwad, Karnataka | KM276436 | KM100018 | KM276280 | - |
|  | *D. latifolia* S | Forestry college, Sirsi, Karnataka | KM276437 | KM100019 | KM276281 | - |
|  | *D. latifolia* T | Ulvi, Anshi National Park | KM276438 | KM100020 | KM276282 | - |
|  | *D. melanoxylon* 1 | Vetaltekadi, Kanchangalli, Pune, Maharashtra | KM276439 | KM100021 | KM276283 | KM276127 |
|  | *D. melanoxylon* 2 | Vetaltekadi, MarutiMandir, Pune, Maharashtra | KM276440 | KM100022 | KM276284 | KM276128 |
|  | *D. melanoxylon* 3 | Vetaltekadi, Pune, Maharashtra | KM276441 | KM100023 | KM276285 | KM276129 |
|  | *D. melanoxylon* 4 | Vetaltekadi, Near parking, Pune, Maharashtra | KM276442 | KM100024 | KM276286 | KM276130 |
|  | *D. melanoxylon* 5 | Vetaltekadi, Pune, Maharashtra | KM276443 | KM100025 | KM276287 | KM276131 |
|  | *D. melanoxylon* 6 | Empress garden, seasonal flower nursery, Pune, Maharashtra | KM276444 | KM100026 | KM276288 | KM276132 |
|  | *D. melanoxylon* 7 | Empress garden, near nala, Pune, Maharashtra | KM276445 | KM100027 | KM276289 | KM276133 |
|  | *D. melanoxylon* 8 | Empress garden parking, Pune, Maharashtra | KM276446 | KM100028 | KM276290 | KM276134 |
|  | *D. melanoxylon* 9 | NCL campus, green house, Pune, Maharashtra | KM276447 | KM100029 | KM276291 | KM276135 |
|  | *D. melanoxylon* 10 | NCL campus, green house, Pune, Maharashtra | KM276448 | KM100030 | KM276292 | KM276136 |
|  | *D. melanoxylon* 11 | NCL campus, green house, Pune, Maharashtra | KM276449 | KM100031 | KM276293 | KM276137 |
|  | *D. melanoxylon* 12 | NCLcampus, post office, Pune, Maharashtra | KM276450 | KM100032 | KM276294 | KM276138 |
|  | *D. melanoxylon* 13 | NCL guest house, Pune, Maharashtra | KM276451 | KM100033 | KM276295 | KM276139 |
|  | *D. melanoxylon* 14 | NCL guest house, Pune, Maharashtra | KM276452 | KM100034 | KM276296 | KM276140 |
|  | *D. melanoxylon* 15 | Biotech department, Pune University, Pune, Maharashtra | KM276453 | KM100035 | KM276297 | KM276141 |
|  | *D. melanoxylon* 16 | Environmental Science department, Pune University, Pune, Maharashtra | KM276454 | KM100036 | KM276298 | KM276142 |
|  | *D. melanoxylon* 17 | Botany department, Pune University, Pune, Maharashtra | KM282580 | KM100037 | KM276299 | KM276143 |
|  | *D. melanoxylon* 18 | Open canteen, Pune University, Pune, Maharashtra | KM282581 | KM100038 | KM276300 | KM276144 |
|  | *D. melanoxylon* 19 | Pune University guest house, Pune, Maharashtra | KM276455 | KM100039 | KM276301 | KM276145 |
|  | *D. melanoxylon* 20 | Lalit kala Kendra, Pune University, Pune, Maharashtra | KM276456 | KM100040 | KM276302 | KM276146 |
|  | *D. melanoxylon* 21 | Fergusson college, Pune, Maharashtra | KM276457 | KM100041 | KM276303 | KM276147 |
|  | *D. melanoxylon* 22 | Amphi theatre, Fergusson college, Pune, Maharashtra | KM276458 | KM100042 | KM276304 | KM276148 |
|  | *D. melanoxylon* 23 | Panhala road, Maharashtra | KM276459 | KM100043 | KM276305 | KM276149 |
|  | *D. melanoxylon* 24 | Panhala road, Maharashtra | KM276460 | KM100044 | KM276306 | KM276150 |
|  | *D. paniculata* 9 | Chinnar Wildlife Sanctuary, Kerala | - | - | KM276307 | KM276158 |
|  | *D. paniculata* 11 | Chinnar Wildlife Sanctuary, Kerala | KM276461 | KM100045 | KM276308 | KM276153 |
|  | *D. paniculata* 12 | Chinnar Wildlife Sanctuary, Kerala | KM276462 | KM100046 | KM276309 | KM276152 |
|  | *D. paniculata* 13 | Chinnar Wildlife Sanctuary, Kerala | KM276463 | KM100047 | KM276310 | KM276154 |
|  | *D. paniculata* 14 | Parambikulam Wildlife Sanctuary, Kerala | KM276464 | KM100048 | KM276311 | KM276151 |
|  | *D. paniculata* 15 | Parambikulam Wildlife Sanctuary, Kerala | KM276465 | KM100049 | KM276312 | KM276159 |
|  | *D. paniculata* 16 | Parambikulam Wildlife Sanctuary, Kerala | KM276466 | KM100050 | KM276313 | - |
|  | *D. paniculata* 17 | Parambikulam Wildlife Sanctuary, Kerala | KM276467 | KM100051 | KM276314 | KM276160 |
|  | *D. paniculata* 18 | Parambikulam Wildlife Sanctuary, Kerala | KM276468 | KM100052 | KM276315 | KM276161 |
|  | *D. paniculata* 19 | KFRI, Kerala | KM276469 | KM100053 | KM276316 | KM276157 |
|  | *D. paniculata* 20 | KFRI, Kerala | KM276470 | KM100054 | KM276317 | KM276155 |
|  | *D. paniculata* 21 | KFRI, Kerala | KM276471 | KM100055 | KM276318 | KM276156 |
|  | *D. paniculata* 22 | KFRI, Kerala | KM276472 | KM100056 | KM276319 | KM276162 |
|  | *D. paniculata* 23 | KFRI, Kerala | KM276473 | KM100057 | KM276320 | KM276163 |
|  | *D. paniculata* 602 | Near Malabar cement factory, Walayar, Palakkad, Kerala | KM276474 | KM100058 | KM276321 | KM276164 |
|  | *D. volubilis* 1 | KFRI, Kerala | KM276522 | - | KM276371 | KM276216 |
|  | *D. volubilis* 2 | KFRI Nilambursubcentre, Kerala | KM276523 | KM100108 | KM276372 | KM276212 |
|  | *D. volubilis* 3 | KFRI Nilambursubcentre, Kerala | KM276524 | KM100109 | KM276373 | - |
|  | *D. volubilis* 5 | KFRI, Kerala | KM276525 | KM100110 | KM276374 | - |
|  | *D. volubilis* 6 | KFRI, Kerala | KM276526 | KM100111 | KM276375 | KM276217 |
|  | *D. volubilis* 9 | Mukkali, Attappady, Palakkad, Kerala | - | KM100112 | - | - |
|  | *D. volubilis* 20 | Quilon, Thenmala, Kerala | KM276527 | KM100113 | KM276376 | KM276218 |
|  | *D. volubilis* 21 | Quilon, Thenmala, Kerala | KM276528 | KM100114 | KM276377 | KM276219 |
|  | *D. volubilis* 22 | Quilon, Thenmala, Kerala | KM276529 | KM100115 | KM276378 | KM276220 |
|  | *D. volubilis* 23 | Kasaragod, Parappa, Kerala | KM276530 | KM100116 | KM276379 | KM276221 |
|  | *D. volubilis* 24 | Kasaragod, Parappa, Kerala | KM276531 | KM100117 | KM276380 | KM276213 |
|  | *D. volubilis* 25 | Kasaragod, Parappa, Kerala | KM276532 | KM100118 | KM276381 | - |
|  | *D. volubilis* 26 | Kurichiad, Wayanad, Kerala | KM276533 | KM100119 | KM276382 | - |
|  | *D. volubilis* 27 | Kurichiad, Wayanad, Kerala | KM276534 | KM100120 | KM276383 | KM276214 |
|  | *D. volubilis* 28 | Kurichiad, Wayanad, Kerala | KM276535 | KM100121 | KM276384 | KM276215 |
|  | *D. volubilis* 29 | KFRI, Kerala | KM276536 | KM100122 | KM276385 | KM276222 |
|  | *D. volubilis* 30 | Kottappara, Ernakulam, Kerala | KM276537 | KM100123 | - | KM276223 |
|  | *D. volubilis* 31 | Naduvathumuzhi, Konni, Kerala | KM276538 | KM100124 | - | KM276224 |
|  | *D. volubilis* 32 | Thannithode-Thekkuthode;Pathanamthitta, Kerala | KM276539 | KM100125 | KM276386 | KM276225 |
|  | *D. volubilis* 33 | Thannithode-Thekkuthode;Pathanamthitta, Kerala | KM276540 | KM100126 | - | - |
|  | *D. lanceolaria* D | Radhanagari, Maharashtra | KM282573 | KM099988 | KM276251 | KM276105 |
|  | *D. lanceolaria* E | Radhanagari, Maharashtra | KM282574 | KM099989 | KM276252 | KM276106 |
|  | *D. lanceolaria* F | Kerle, Kolhapur, Maharashtra | KM282575 | KM099990 | KM276253 | KM276110 |
|  | *D. lanceolaria* G | Kerle, Kolhapur, Maharashtra | KM282576 | KM099991 | KM276254 | KM276111 |
|  | *D. lanceolaria* CRD | Canal road, Pune, Maharashtra | KM282577 | KM099992 | KM276255 | KM276107 |
|  | *D. lanceolaria* DIRC | DIRC, NCL campus, Pune, Maharashtra | KM282578 | KM099993 | KM276256 | KM276109 |
|  | *D. lanceolaria* KERALA | Kerala | KM282579 | KM099994 | KM276257 | KM276108 |
|  | *D. sissoo* 1 | ARAI tekdi, Pune, Maharashtra | KM276496 | KM100080 | KM276343 | KM276184 |
|  | *D. sissoo* 2 | Bavdhan road, Pune, Maharashtra | KM276497 | KM100081 | - | KM276185 |
|  | *D. sissoo* 3 | Empress garden, Pune, Maharashtra | - | - | KM276344 |  |
|  | *D. sissoo* 4 | Bhandarkar road, Pune, Maharashtra | KM276498 | KM100082 | KM276345 | KM276186 |
|  | *D. sissoo* 5 | Pravrangar, Maharashtra | KM276499 | KM100083 | KM276346 | KM276187 |
|  | *D. sissoo* 6 | Panchavati area, Pune, Maharashtra | KM276500 | KM100084 | KM276347 | KM276188 |
|  | *D. sissoo* 7 | BARC colony, Mumbai, Maharashtra | KM282582 | KM100085 | KM276348 | KM276189 |
|  | *D. sissoo* 8 | Maharashtra Cultural Centre garden, Dharavi, Mumbai, Maharashtra | KM276501 | KM100086 | KM276349 | KM276190 |
|  | *D. sissoo* 9 | Parks and Gardens, BARC, Mumbai, Maharashtra | KM276502 | KM100087 | KM276350 | KM276191 |
|  | *D. sissoo* 10 | Maharashtra Cultural Centre garden, Dharavi, Mumbai, Maharashtra | KM282583 | KM100088 | KM276351 | KM276192 |
|  | *D. sissoo* 11 | Pasaranighat, Mahableshwar, Maharashtra | KM276503 | KM100089 | KM276352 | KM276193 |
|  | *D. sissoo* 12 | Pasaranighat, Mahableshwar, Maharashtra | KM276504 | KM100090 | KM276353 | KM276194 |
|  | *D. sissoo* 13 | Gadhinglaj, Dist. Kolhapur, Maharashtra | KM276505 | KM100091 | KM276354 | KM276195 |
|  | *D. sissoo* 14 | Atith, NH4 highway, Maharashtra | KM276506 | KM100092 | KM276355 | KM276196 |
|  | *D. sissoo* 15 | Radhanagari Road, Maharashtra | KM276507 | KM100093 | KM276356 | KM276197 |
|  | *D. sissoo* 16 | Jotiba road, Kolhapur, Maharashtra | KM276508 | KM100094 | KM276357 | KM276198 |
|  | *D. sissoo* 17 | Jotiba road, Kolhapur, Maharashtra | KM276509 | KM100095 | KM276358 | KM276199 |
|  | *D. sissoo* 18 | Lalbagh garden, Banglore, Karnataka | KM276510 | KM100096 | KM276359 | KM276200 |
|  | *D. sissoo* 19 | Ranganthittu, Mysore, Karnataka | KM276511 | KM100097 | KM276360 | KM276201 |
|  | *D. sissoo* 20 | Mysore University Guest House, Karnataka | KM276512 | KM100098 | KM276361 | KM276202 |
|  | *D. sissoo* 21 | Mysore University, Karnataka | KM276513 | KM100099 | KM276362 | KM276203 |
|  | *D. sissoo* 22 | Aurangabad, Maharashtra | KM276514 | KM100100 | KM276363 | KM276204 |
|  | *D. tamarindifolia* 1 | Sahasralinga, Karnataka | KM276515 | KM100101 | KM276364 | KM276205 |
|  | *D. tamarindifolia* 2 | Sahasralinga, Karnataka | KM276516 | KM100102 | KM276365 | KM276206 |
|  | *D. tamarindifolia* 3 | Kumta, Karnataka | KM276517 | KM100103 | KM276366 | KM276207 |
|  | *D. tamarindifolia* 4 | Nuggi nursery, Anshi National Park, Goa | KM276518 | KM100104 | KM276367 | KM276208 |
|  | *D. tamarindifolia* 5 | Nuggi nursery, Anshi National Park, Goa | KM276519 | KM100105 | KM276368 | KM276209 |
|  | *D. tamarindifolia* 6 | Nuggi nursery, Anshi National Park, Goa | KM276520 | KM100106 | KM276369 | KM276210 |
|  | *D. tamarindifolia* 7 | Nuggi nursery, Anshi National Park, Goa | KM276521 | KM100107 | KM276370 | KM276211 |
|  | *D. horrida* A | Gaganbavada, Maharashtra | KM276404 | KM099979 | KM276242 | KM276096 |
|  | *D. horrida* 1 | Koyananagar, Maharashtra | KM276405 | KM099980 | KM276243 | KM276097 |
|  | *D. horrida* 3 | Hiranyakeshi temple bridge Amboli, Maharashtra | KM276406 | KM099981 | KM276244 | KM276098 |
|  | *D. horrida* 4 | Hiranyakeshi temple, Amboli, Maharashtra | KM276407 | KM099982 | KM276245 | KM276099 |
|  | *D. horrida* 5 | Hiranyakeshi temple, Amboli, Maharashtra | KM276408 | KM099983 | KM276246 | KM276100 |
|  | *D. horrida* 6 | Nangartas waterfall, Amboli, Maharashtra | KM276409 | KM099984 | KM276247 | KM276101 |
|  | *D. horrida* 7 | Anshi National Park, Goa | KM276410 | KM099985 | KM276248 | KM276102 |
|  | *D. horrida* 8 | Anshi National Park, Goa | KM276411 | KM099986 | KM276249 | KM276103 |
|  | *D. horrida* 9 | Nuggi nursery, Anshi National Park, Goa | KM276412 | KM099987 | KM276250 | KM276104 |

‘-’ indicates the sample that did not generate good and readable DNA sequence and therefore not deposited in NCBI database
