# Supplementary material for: Two New Potential Barcodes to Discriminate Dalbergia Species
Source: PLoS One. 2015 Nov 16;10(11):e0142965. doi: 10.1371/journal.pone.0142965 (PMC4646644; doi:10.1371/journal.pone.0142965)
Supplement: S2 Dataset — Primers used in DNA barcoding of Dalbergia species. (DOCX) [file pone.0142965.s004.docx]

**S2 Dataset**. **Primer details.** Primers used in DNA barcoding of *Dalbergia* species

| **Locus** | **Primer name** | **Primer sequence (5’→3’)** | **Approximate size in genus *Dalbergia*** | **Reference** |
| --- | --- | --- | --- | --- |
| *matK* | matK2.1a F | ATC CAT CTG GAA ATC TTA GTT C | 900 bp | Royal Botanic Gardens, Kew |
|  | matK3.2 R | CTT CCT CTG TAA AGA ATT C |  |  |
| *rbcL* | rbcL a F | ATG TCA CCA CAA ACA GAG ACT AAA GC | 650 bp | Fazekas et al. 2009 [3] |
|  | rbcL ajf634 R | GAA ACG GTC TCT CCA ACG CAT |  |  |
| *trnH-psbA* | trnH-psbA F | CGC GCA TGG TGG ATT CAC AAT CC | 300 bp | Kress et al. 2005 [6] |
|  | trnH-psbA R | GTT ATG CAT GAA CGT AAT GCT C |  |  |
| *nrITS* | nrITS 5aF | CCT TAT CAT TTA GAG GAA GGA G | 700 bp | Chen et al. 2010 [12] |
|  | nrITS 4R | TCC TCC GCT TAT TGA TAT GC |  |  |
