# Supplementary material for: Two New Potential Barcodes to Discriminate Dalbergia Species
Source: PLoS One. 2015 Nov 16;10(11):e0142965. doi: 10.1371/journal.pone.0142965 (PMC4646644; doi:10.1371/journal.pone.0142965)
Supplement: S3 Dataset — PCR conditions for matK, rbcL, trnH-psbA and nrITS (DOCX) [file pone.0142965.s005.docx]

**S3 Dataset.** **PCR reaction details.** PCR conditions for *matK*, *rbcL*, *trnH-psbA* and *nrITS*

|  | **Effective concentration of various components** | | | | | | | | | **Cycling conditions** | |
| --- | --- | --- | --- | --- | --- | --- | --- | --- | --- | --- | --- |
| **Locus** | **DNA (ng)** | **dNTPs (mM)** | **Buffer** | **MgCl_2_**  **(mM)** | **Forward primer (pmoles)** | **Reverse primer (pmoles)** | **DMSO**  **(%)** | ***Taq* DNA polymerase**  **(units)** | **Total reaction volume (µL)** | **Details of steps** | **No. of cycles** |
| *rbcL* | 50-60 | 0.2 | 10X | 1.0 | 0.16 | 0.16 | 4.0 | 0.048 | 20 | Initial denaturation at 95°C for 2 min, cycle denaturation at 95°C for 30 sec, annealing at 64°C for 45 sec, extension at 72°C for 50 sec and final extension at 72°C for 5 min | 35 |
| *trnH*-*psbA* | 60-70 | 0.2 | 10X | 1.0 | 0.16 | 0.16 | - | 0.048 | 25 | Initial denaturation at 94°C for 5 min, cycle denaturation at 94°C for 1 min, annealing at 65°C for 30 sec, extension at 72°C for 1 min and final extension at 72°C for 5 min |  |
| *nrITS* | 60-70 | 0.2 | 10X | 0.5 | 0.16 | 0.16 | 4.0 | 0.144 | 25 | Initial denaturation at 94°C for 5 min, cycle denaturation at 94°C for 1 min, annealing at 56°C for 30 sec, extension at 72°C for 1 min and final extension at 72°C for 5 min |  |
| *matK* | 50-60 | 0.2 | 10X | 1.0 | 0.12 | 0.12 | - | 0.048  (AccuTaq LA DNA polymerase) | 25 | Initial denaturation at 94°C for 3 min, cycle denaturation at 94°C for 30 sec, annealing at 48°C for 30 sec, extension at 68°C for 1 min and final extension at 68°C for 2 min |  |
